# Methods and Designs of Modern Breast Cancer Confirmatory Trials

**DOI:** 10.3390/cancers13112757

**Published:** 2021-06-02

**Authors:** Julien Péron, Thibaut Reverdy, Colette Smenteck, Marion Cortet, Benoît You, Gilles Freyer

**Affiliations:** 1 Service d’Oncologie Médicale, Institut de Cancérologie des Hospices Civils de Lyon, 69002 Lyon, France; thibaut.reverdy@chu-lyon.fr (T.R.); benoit.you@chu-lyon.fr (B.Y.); gilles.freyer@chu-lyon.fr (G.F.); 2 Laboratoire de Biométrie et Biologie Evolutive, Equipe Biostatistique-Santé, CNRS UMR 5558, Université Claude Bernard Lyon 1, 69100 Villeurbanne, France; 3 Faculté de Médecine et de Maïeutique Lyon-Sud-Charles Mérieux, Université Claude Bernard Lyon 1, 69310 Pierre-Bénite, France; 4 Laboratoire Parcours Santé et Systémique, EA 4129, Université Claude Bernard Lyon 1, 69002 Lyon, France; colettesmentek@yahoo.fr; 5 Service de gynécologie, Hôpital de la Croix Rousse, Institut de Cancérologie des Hospices Civils de Lyon, 69002 Lyon, France; marion.cortet@chu-lyon.fr

**Keywords:** randomized clinical trials, statistical methods, endpoints, trial design

## Abstract

**Simple Summary:**

The benefit–risk assessments of new drugs for breast cancer (BC) face several challenges, as all stakeholders do not agree on the evidence bar required for market authorization, and by the fragmentation of breast cancer diagnosis. In this study, we describe the methods and designs of breast cancer confirmatory trials published between 2001 and 2020. We found that the quality of the evidence supporting new breast cancer drugs was improving over time, but that patient-relevant endpoints such as survival and quality of life remained unfrequently used as primary endpoints.

**Abstract:**

Background: The benefit–risk assessments of new drugs for breast cancer (BC) face several challenges, as all stakeholders do not agree on the evidence bar required for market authorization, and by the fragmentation of breast cancer diagnosis. The aim of this study was to describe the changes in methods and designs of breast cancer confirmatory trials. Methods: All phase III randomized trials published between 2001 and 2020 and assessing systemic BC therapies were included. Trials’ main characteristics, endpoints, and statistical methods were collected using a standardized data extraction form. Results: A total of 347 randomized controlled trials (RCTs) met the inclusion criteria. While most older trials (79%) included all subtypes of breast cancer, most recent trials populations were limited to one large intrinsic BC subgroup (69%). The use of gatekeeping testing strategies increased dramatically from 9% to 71%. The use of overall survival (OS) as an endpoint in the trials increased over time, but its use as a primary endpoint remained infrequent. The inclusion of OS testing in a hierarchical sequence in case of positive testing of a tumor-centered or composite endpoint appeared to have become the new standard. Conclusion: Our findings indicate some improvements in the quality of the evidence-base supporting new breast cancer drugs. The rigorous assessment of patient-relevant endpoints has increased over time, but this improvement is mainly related to the analysis of OS as a secondary endpoint analyzed in a hierarchical sequence.

## 1. Introduction

Breast cancer (BC) is the most common malignancy diagnosed in women worldwide, and so the development of new drugs and new therapeutic strategies is a public health priority. Health Technology Assessment (HTA) bodies have published regulatory requirements for efficacy and safety assessment of new treatments [1]. The use of patient-relevant clinical endpoints, such as overall survival, quality of life or patient-reported outcomes for assessing the new drugs’ benefit–risk has regularly been emphasized by most HTA bodies and international scientific societies [2,3]. Such an aim requires at least one high-quality randomized placebo-controlled trial investigating a clinical endpoint of interest, such as overall survival (OS), as a primary endpoint. Moreover, OS assessment requires a long follow-up in order to demonstrate the long-term effect of the experimental regimen. 

Such an ideal drug development strategy faces in practice two main limits. First, many oncology stakeholders advocate that it may not be ethical to perform placebo-controlled trial, and to wait for long-term assessment of treatment effect before granting patient access to promising drugs. In reaction to the HIV crisis, the principle of accelerated approval of drugs has been adopted at various level around the world, allowing for approval based on either a surrogate endpoint reasonably likely to predict clinical benefit, or an intermediate endpoint in the direct causal pathway of a more meaningful endpoint. In oncology, the use of tumor-centered surrogate or intermediate endpoints have led to faster access to the market, but these endpoints have inconsistent clinical relevance. It resulted in a wide distribution in the therapeutic benefits associated with approved anticancer drugs, suggesting a similarly wide variation in the value that they bring to society [4,5]. Consequently, there have been calls to raise the evidence bar for market authorization of new cancer drugs [6,7,8]. The second main challenge is the advent of personalized medicine in oncology, through the identification of potent molecularly targeted agents for patients with tumors bearing specific molecular alterations. Even for a disease as frequent as breast cancer, the increasing fragmentation of breast cancer patients could be prone to challenge the feasibility of large-size confirmatory trials.

In this evolving context, the primary objective of this study was to describe the changes in methods and designs of confirmatory trials involving breast cancer patients, in order to discuss future perspectives. 

## 2. Materials and Methods

### 2.1. Study Selection

We searched MEDLINE via PubMed to identify all publications of RCTs assessing systemic anticancer therapies for breast neoplasms published between January 2001 and December 2020 in a representative sample of 12 journals that are thought to publish the majority of breast cancer RCTs: Annals of Oncology; British Journal of Cancer; Breast Cancer Research and Treatment; Cancers; European Journal of Cancer; JAMA; JAMA oncology; Journal of Clinical Oncology; Journal of the National Cancer Institute; Lancet; Lancet Oncology; and New England Journal of Medicine. Exclusion criteria were pediatric studies; treatment solely with radiotherapy or surgery; phase I, II, or IV trials; meta-analyses, overviews, or publications using pooled data from two or more trials; secondary reports of previously published trials. 

### 2.2. Definition of Trial Characteristics

A standardized data extraction form was developed by two authors (J.P. and T.R.) to capture all data reported in this review. The definition of each item was discussed and validated by consensus prior to data collection. Trials were considered industry-funded if there was at least partial funding by an industry identified in the publication. The geographic regions where RCTs were led were derived from the addresses of the first author institutions. The population of interest ‘large intrinsic subgroup’ was defined as one of the following categories: HER2-positive BC, triple negative BC, estrogen receptors (ER)-positive breast cancer. Coprimary endpoints were defined as more than one non-hierarchical endpoint involved in the statistical testing strategy. Hierarchical analyses (or gatekeeping analyses) were defined as sequential statistical tests where lower-level endpoints were tested only if the statistical test of higher priority endpoint was positive. Composite endpoints were defined as time-to-event endpoints based on a combination of individual events, including death and at least one other event related to the tumor evolution. Tumor-centered endpoints included all measurements of tumor evolution (including radiological assessment, biological, or histological markers evolution). PROs were defined as any report of the status of a patient’s health condition that came directly from the patient, without interpretation of the patient’s response by a clinician or anyone else. PROs included mainly symptoms and health related QoL assessments. 

### 2.3. Statistical Analysis

Continuous variables were described using their median values, and interquartile ranges (IQR). Binary variables were described using proportions. Cochran–Armitage, Cochran–Mantel–Haenzel, or Jonckheere–Terpstra tests for trend were used to identify a measurable change in reporting frequency or distribution of continuous variables over the years, as appropriate. For continuous covariable evolution, univariate linear regression models were used. As the number of missing values was very low, it was handled by listwise deletion. Statistical analyses and illustrations were performed using R Software v3.5.1 (http://www.R-project.org/, (accessed on 23 May 2021)).

## 3. Results

### 3.1. Characteristics of Selected RCTs

**Figure 1:**
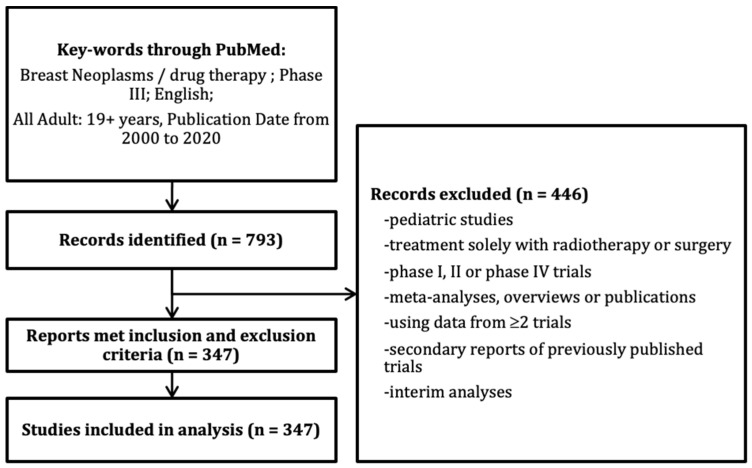
Selection of randomized clinical trials in the systematic review.

From the 793 trials initially screened, a total of 347 RCTs met the inclusion criteria (Figure 1). Overall, 76% of trials were at least partially funded by the pharmaceutical industry (Table 1). This proportion was stable over time. Most RCTs were published in currently high IFs journals (>10). Most trials were led by a European country, and this proportion decreased over time while the number of trials led by an Asian country increased. Cytotoxic chemotherapy was the most frequent class of experimental treatment, but this proportion decreased from 64% in the period 2001–2005 to 33% in those 2016–2020, while the proportion of trials that investigated a targeted therapy (including hormonal therapies) increased from 6% to 44% in the same time windows (P < 0.0001).

### 3.2. Trial Designs

In the assessed inclusion period, the proportion of trials in the metastatic setting decreased from 64% in the period 2001–2005 to 38% in the period 2016–2020 (P = 0.0014) and the proportions of trials in the metastatic and early-stage settings were similar. The vast majority of trials were superiority trials, and the proportion of non-inferiority or equivalence trials remained constant over time. The proportion of trials with more than two randomized groups was below 10%, and constant over time. A change in the assessed trials’ populations of interest was observed. While most trials in the first time-period included all breast cancers without any limitation to any given biological subgroup, the majority of trials published between 2016 and 2020 focused on one large intrinsic subgroup (ER positive–HER2-negative BC; HER2-positive BC; or triple negative BC). In the last years, some trials recruited a narrower biologically defined population of breast cancer patients (population smaller than one of the large intrinsic subgroup), but this was still limited to a small proportion of trials (4% in the last time-period). While the included populations tend to be more and more restricted to biologically defined subgroups of breast cancer patients, the overall sample size of trials increased over time (Table 2). In both metastatic and early breast cancer stage trials, most trials used an active control (+/− placebo) as a comparator, and this proportion remained stable over time (Table 3). In the metastatic setting, most trials included patients in the first-line of treatment at the advanced stage. For adjuvant/neoadjuvant trials, the experimental strategy was given on the same time period as the comparator, and the proportion of trials investigating a shorter treatment duration were infrequent (12% over all time-periods), while 32% investigated a longer treatment duration. A large proportion of trials investigated the addition of a new treatment to the standard treatment (47%), while 37% investigated the replacement of the standard treatment by the experimental one. These proportions were constant over time (Table 3).

### 3.3. Statistical Analysis Procedures

The use of gatekeeping or hierarchical testing strategies increased dramatically during the period of observations, from 9% among older trials to 71% during the most recent time-period. Among the 146 trials including a hierarchical analysis, 117 (80%) included an OS analysis in the non-first priority tests, while 19 (13%) assessed the primary endpoint in a patient population other than the primary analysis population. The use of two or more co-primary endpoints, with a split of the type-1 error between the two or more tests remained infrequent though all time-periods (Table 2).

### 3.4. Trial Endpoints

**Figure 2:**
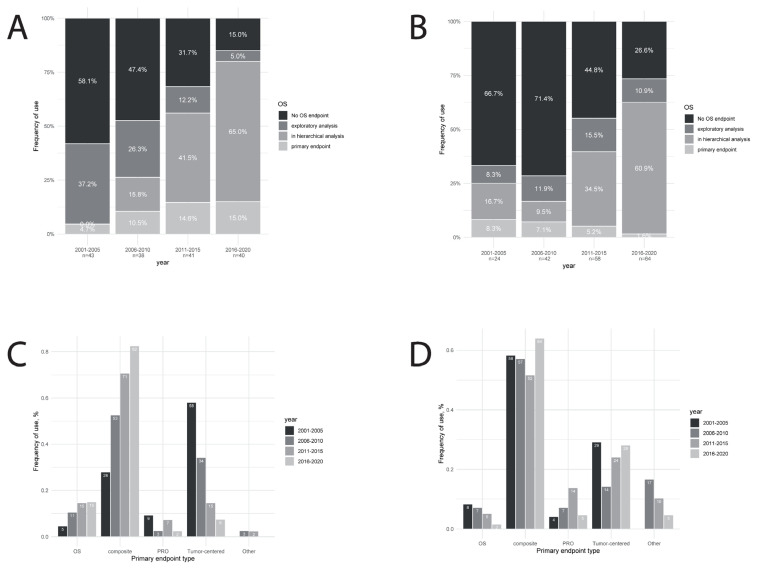
Use of overall survival over time in (**A**) metastatic or advanced breast cancer trials and (**B**) early-stage breast cancer trials. Type of primary endpoints used in (**C**) metastatic or advanced breast cancer trials and (**D**) early-stage breast cancer trials. Footnotes: OS = overall survival; PRO = patient-reported outcomes.

The use of OS as an endpoint in the trials increased over time, in both the metastatic setting (from 42% to 85%, Figure 2A) and in the early-stage breast cancer setting (from 33% to 83%, Figure 2B). The use of OS as a primary endpoint remained infrequent (from 5% to 15% in the metastatic setting, P = 0.11, and from 8% to 16% in the early-stage setting, P = 0.11). On the other hand, the use of OS in a hierarchical sequence appeared to become a new standard in most recent trials (from 0% to 68% in the metastatic setting, P < 0.0001, and from 17% to 61% in the early-stage setting, P < 0.0001). Tumor-centered endpoints such as response rate or pathological response were the most frequent primary endpoints among metastatic trials during the first time-period, but the proportion decreased over time (from 58% to 8%, P < 0.0001, Figure 2C). In the recent trials, the most frequent type of primary endpoint was composite endpoints such as progression-free survival or disease-free survival (from 28% to 83%, %, P < 0.0001, Figure 2C), while OS and patient-reported outcomes (PRO) remained infrequently used. In early-stage breast cancer trials, this evolution was not observed regarding the type of endpoint used as primary endpoint (Figure 2D), and composite endpoints remained the most frequently used primary endpoints over all time-periods. The use of OS as a primary endpoint was even decreasing over time (from 8% to 2%, P = 0.11).

## 4. Discussion

To our knowledge, this is the first large-scale study describing the designs and methods of breast cancer confirmatory trials during the first two decades of the 21st century. These results illustrate the evolving landscape of breast cancer treatment, but also the new paradigms of benefit–risk assessment of new treatments mostly driven by major HTA bodies, international scientific societies, and pharma industries [9,10]. The growing number of published confirmatory trials and the increasing sample size highlight the sustained investment and interest in this field of research. Most recent trials included only one of the main breast cancer subtypes defined by endocrine-receptor status and HER2 status. This observation is a logical consequence of the recent advances in the therapeutic management of patients with breast cancer including the development of anti-HER2 therapies, potent endocrine therapies, and CDK4/6 inhibitors. Trials focusing on a biologically defined population narrower than one of the large intrinsic subgroups remained surprisingly low even in the most recent period. Most recent advances in the biological stratification of breast cancers are then unlikely to be translated in the short term into new biology-driven therapeutic decision-making. While the increased knowledge in tumor and immune system biology might have led to trials hypothesizing large and clinically relevant treatment effect in smaller patients subgroups, a recent investigation reported that contemporary cancer trials are still designed to identify small arguably relevant treatment effects [11]. This observation is in line with our findings of an increase over time in the trials’ sample sizes.

While composite endpoint, such as progression-free survival (PFS) in the metastatic setting or disease-free survival (DFS) in the early stage setting have a recognized clinical relevance [12], they are not recognized as the most appropriate indicators of treatment benefit [1]. Composite endpoints are then to be considered as intermediate endpoints that could be used to replace OS, but they should be formally validated as surrogates. The use of non-validated surrogate endpoints as primary endpoints in confirmatory trials may result in approved drugs with questionable benefits, but frequently side effects (and invariably high costs). With the notable exception of DFS for HER2-positive early-stage breast cancer [13], composite endpoints have shown some correlation with OS at the individual level but have failed to demonstrate any consistent strong correlation between treatment effect on the candidate surrogate and treatment effect on OS at the trial level [14,15,16].

Overall survival is still considered the gold standard primary endpoint as it is a meaningful and relevant clinical outcome with a low risk of biased assessment. However, there are limitations to the use of OS as the primary endpoint in both metastatic and early-stage setting. There are multiple available effective therapies for the treatment of metastatic BC and patients generally received sequential effective treatments after failure of drugs used in a given clinical trial, with the potential for a long post-treatment survival period. Under this scenario, it has been advocated that it became more difficult to demonstrate OS gain [17]. However, this assumption has been challenged by the OS advantage recently reported with CDK4-6 inhibitors [18,19] for advanced ER-positive breast cancer despite the availability of multiple post-progression therapies. For early-stage disease, the analysis of overall survival requires a very long follow-up, implying a delayed access to innovative treatment for patients if a demonstration of an OS benefit was mandatory for the evaluation by HTA bodies.

The review of most recent clinical trials in our study shows that the OS has been very infrequently used as a primary endpoint, and there was no sign of any relevant increase in its use over time. However, a compromise between OS and intermediate endpoints seems to have been reached with the increasing use of hierarchical analyses including the analysis of OS with an appropriate control of the type-1 error if the statistical test of one or more higher priority endpoints were statistically positive.

The use of tumor-centered endpoints as primary endpoints (such as response rate in the metastatic setting and pathological complete response in the early-stage setting) have been less frequent in the metastatic setting, while their use remained stable in the early-stage setting. The increasing adoption of composite endpoint over tumor-centered endpoints for metastatic disease is probably linked to the perceived good clinical value of PFS [12], since similar poor surrogate properties have been reported in 2008 for response rate and progression-free survival [20]. Both Food and Drugs administration (FDA) and European Medical Agency (EMA) have developed accelerated assessment programs to facilitate earlier authorizations. Oncology is from far the main domain concerned by accelerated assessment programs, as there is often unmet medical need and the public health issue is often characterized. Under these programs, drugs could be authorized on the basis of early evidence of efficacy and safety, based from single-arm trials and/or surrogate endpoints. A recent review pointed out that only 55% accelerated approvals were later converted to regular approval, while most of the remaining confirmatory approvals were still pending [21]. Similar findings were reported when focusing on all drugs approved via the accelerated FDA approval pathway [22].

The development of bevacizumab is a good illustration since it was granted accelerated approval in the US for the treatment of metastatic breast cancer based on improvements in PFS, and it was revoked when subsequent confirmatory trials failed to demonstrate a benefit in OS but did demonstrate a moderate increase in toxic effects [23]. To avoid such scenario in the future, possible strategies would be to validate more surrogate measures, when the use of OS as a primary endpoint is deemed unpractical, or to reach consensus on patient-relevant outcomes that can be captured earlier than OS, such as long-term tumor control, patient-reported outcomes (PRO) or health-related quality of life (HR-qol). While HR-qol is universally recognized as an important objective, especially in the metastatic setting, it remained rarely used as a primary endpoint in this review, even when considering hierarchical analyses. Considering the subjective nature of HR-qol data, the complexity of its analysis given its multidimensional structure, and the difficulty to separate impact on HR-qol of disease-related symptoms from treatment toxicity, a lot of work has been done to optimize data collection, to standardize questionnaire, analysis and reporting [24]. Given the increasing acceptance of PROs and HR-qol as important endpoints by all stakeholders [25], it might be time to go one step forward and to implement HR-QoL or PRO as co-primary endpoints or to include such endpoints in the hierarchical analyses of breast cancer confirmatory trials, at least in the metastatic setting. Even with a robust demonstration of HR-QoL or PRO improvement, there must be confidence that the observed HR-QoL or PRO benefit is achieved without any reduction in efficacy. For this reason, it seems premature to recommend the use of such endpoint as single primary endpoint.

In all other scenarios where a demonstration for an OS benefit is expected to demonstrate the positive benefit–risk balance of a new product, the early approval of new drugs based on intermediate endpoint should probably be limited to situations where the benefit observed on the intermediate endpoint is of a magnitude and of a clinical relevance that is deemed sufficient to anticipate an OS benefit with a high probability, and where the research plan is designed to provide in the future a methodologically robust assessment of the treatment effect on OS [4]. During older time-periods, only a minority of confirmatory trials included OS or quality of life as a primary analysis, or in any hierarchical analysis involving a control of the type-1 error. In the most recent time-period, OS remained barely used as a primary endpoint, but it was most often included as a secondary endpoint in a hierarchical sequence. This analysis strategy has the potential to allow early access to the market through accelerated approval procedures, with a subsequent confirmatory assessment of the benefit based on OS. The observation that a robust demonstration of an OS benefit is available for most recent trials is reassuring, while the reluctance to use it as primary endpoint, even in the metastatic setting, is more troubling. This might be linked to the importance of early access to the market from the point of view of the pharma industry, clinicians, and patients.

While the stratification of early-stage breast cancers according to clinical characteristics, stage or biological tumor characteristics enabled the identification of good prognosis subgroups [26,27], the proportion of trials investigating an experimental treatment with a shorter duration remained low over the years, as was the proportion of trials with no new investigational drugs (de-escalation trials). De-escalation trials are welcomed by the oncology community as they are seen as a way to overcome current limitation of systemic treatment for early-stage breast cancer including overtreatment of patients already cured by locoregional therapy, short- and long-term toxicities, and increasing costs [28]. The small proportion of such trials might be explained by the relative underfunding of such trials, given the lack of interest from the pharmaceutical industry and the lack of sufficient purely academic funding.

The current study has a number of limitations. Our analysis was limited to studies published in leading medical journals of the field, and therefore is potentially subject to publication bias. As we focused on confirmatory trials in an important medical field, it is unlikely that the inclusion of unpublished trials and of trials published in other journals would have changed our findings significantly. If any change would be expected with the inclusion of such trials, it would be in the direction of a lower overall quality of the methods. We did not evaluate the trials protocols and relied on reported methods and results, with a known heterogeneity in the reporting quality [29]. Additionally, a single investigator rated most publications, which could have introduced subjectivity into the process. To reduce subjectivity, a data collection template was designed and piloted before its use for data collection in this study.

## 5. Conclusions

In conclusion, our findings indicate some improvements in the quality of the evidence-base supporting new breast cancer drugs. This description of the evolving landscape in breast cancer confirmatory trials is interesting and should be a good support to consider the future directions that we want to take as a community. The rigorous assessment of patient-relevant endpoints has clearly increased over time, but the analysis of OS has essentially remained a secondary endpoint analyzed in hierarchical sequences. PRO and HR-qol remain mainly exploratory endpoints, when they are included in the trials.

## Figures and Tables

**Table 1:** Trial characteristics (*n* = 347).

Study Characteristics		Studies	
		*n*	%
**Year of publication**	2001–2005	67	19
	2006–2010	80	23
	2011–2015	96	28
	2016–2020	104	30
**Tumor setting**	Localized	184	53
	Advanced or metastatic	158	46
	Both	5	1
**Sources of trial funding (NA = 7)**	Government/Foundation	81	24
	Funded by industry	259	76
**Journal**	Journal of Clinical Oncology	134	39
	Annals of Oncology	41	18
	New England Journal of Medicine	32	9
	Lancet/Lancet Oncol	58	17
	European Journal of Cancer	19	6
	Other journals	63	18
**Regions in which RCTs were led**	North America	95	27
	Europe	200	57
	Asia	27	8
	Other	25	7
**Investigational therapy**	Cytotoxic chemotherapy	155	45
	Hormonal therapy	59	17
	Molecular targeted therapy	96	28
	Immunotherapy	2	1
	Other	35	10

Footnotes: RCT = randomized controlled trial.

**Table 2:** Trial methods over time, all trials (*n* = 347).

Study Characteristics	2001–2005, *n* = 67	2006–2010, *n* = 80	2011–2015, *n* = 96	2016–2020, *n* = 104	P
**Non-inferiority design**	13 (20%)	2 (3%)	9 (10%)	16 (16%)	0.96
**Population of interest**					<0.0001
All BCs	52 (79%)	56 (70%)	50 (52%)	28 (27%)	
Large intrinsic subgroup	14 (21%)	24 (30%)	45 (47%)	72 (69%)	
Narrower biologically defined subgroup	0 (0%)	0 (0%)	1 (1%)	4 (4%)	
**Inclusion period length in months, median (IQR)**	37 (25–49)	37 (29–56)	39 (29–60)	32 (21–51)	0.27
**Sample size, median (IQR)**	375 (258–600)	532 (250–1027)	602 (299–1157)	682 (461–1534)	<0.0001
**>2 treatment groups**	2 (3%)	9 (11%)	7 (7%)	6 (6%)	0.91
**Coprimary endpoint (type-1 error split)**	2 (3%)	3 (4%)	7 (7%)	7 (7%)	0.54
**Hierarchical analysis**	6 (9%)	22 (28%)	44 (46%)	74 (71%)	<0.0001
Other analysis population	0 (0%)	6 (2%)	6 (2%)	7 (2%)	0.097
Other endpoint except OS	2 (3%)	6 (8%)	12 (13%)	26 (25%)	0.00012
OS endpoint	4 (6%)	10 (13%)	37 (39%)	66 (63%)	<0.0001

Footnotes: BC = breast cancer; IQR = interquartile range.

**Table 3:** Trial strategy over time according to clinical setting.

	Metastatic Trials (*n* = 162)				
Study Characteristics	2001–2005, *n* = 43	2006–2010, *n* = 38	2011–2015, *n* = 41	2016–2020, *n* = 40	P
**Control arm**					0.47
Active treatment +/−placebo	38 (88%)	37 (97%)	37 (90%)	34 (85%)	
Placebo+BSC/observation	3 (7%)	0	2 (5%)	5 (13%)	
BSC/observation	2 (5%)	1 (3%)	2 (5%)	1 (3%)	
**Treatment line**					0.94
1st only	27 (63%)	24 (63%)	22 (54%)	23 (58%)	
1st and more	9 (21%)	7 (18%)	9 (22%)	8 (20%)	
2nd and more	6 (14%)	5 (13%)	9 (22%)	6 (15%)	
3rd and more	1 (2%)	2 (5%)	1 (2%)	3 (8%)	
	**Early breast cancer trials (*n* = 188)**				
**Study characteristics**	**2001–2005, *n* = 24**	**2006–2010, *n* = 42**	**2011–2015, *n* = 58**	**2016–2020, *n* = 64**	**P**
**Control arm**					0.098
Active treatment +/−placebo	22 (82%)	31 (74%)	46 (79%)	50 (78%)	
Placebo+BSC/observation	2 (8%)	9 (21%)	4 (7%)	6 (9%)	
BSC/observation	0 (0%)	2 (5%)	8 (14%)	8 (13%)	
**Experimental treatment duration vs. standard**					0.14
Shorter					
Same	4 (17%)	3 (8%)	2 (4%)	12 (19%)	
Longer	12 (52%)	20 (51%)	35 (65%)	33 (53%)	
	7 (30%)	16 (41%)	17 (32%)	17 (27%)	
**Experimental strategy**					0.45
Addition of a new drug to standard	11 (46%)	20 (48%)	30 (52%)	28 (44%)	
Replacement of a standard drug	9 (38%)	19 (45%)	16 (28%)	26 (41%)	
No new drug	4 (17%)	3 (7%)	10 (21%)	10 (16%)	

Footnotes: BSC = Best Supportive Care.
